# Identifying transcriptomic profiles in ovine spleen after repetitive vaccination

**DOI:** 10.3389/fimmu.2024.1386590

**Published:** 2024-07-12

**Authors:** Aitor Guisasola-Serrano, Martin Bilbao-Arribas, Endika Varela-Martínez, Naiara Abendaño, Marta Pérez, Lluís Luján, Begoña Marina Jugo

**Affiliations:** ^1^ Genetics, Physical Anthropology and Animal Physiology Dpt., Faculty of Science and Technology, University of the Basque Country (UPV/EHU), Leioa, Spain; ^2^ Animal Pathology Dpt., Faculty of Veterinary, University of Zaragoza, Zaragoza, Spain

**Keywords:** vaccination, aluminum hydroxide, transcriptome, RNA-seq, sheep, spleen

## Abstract

Aluminum hydroxide has long been employed as a vaccine adjuvant for its safety profile, although its precise mechanism of action remains elusive. In this study, we investigated the transcriptomic responses in sheep spleen following repetitive vaccination with aluminum adjuvanted vaccines and aluminum hydroxide alone. Notably, this work represents the first exploration of the sheep spleen transcriptome in such conditions. Animals were splitted in 3 treatment groups: vaccine group, adjuvant alone group and control group. A total of 18 high-depth RNA-seq libraries were sequenced, resulting in a rich dataset which also allowed isoform-level analysis. The comparisons between vaccine-treated and control groups (V vs C) as well as between vaccine-treated and adjuvant-alone groups (V vs A) revealed significant alterations in gene expression profiles, including protein coding genes and long non-coding RNAs. Among the differentially expressed genes, many were associated with processes such as endoplasmic reticulum (ER) stress, immune response and cell cycle. The analysis of co-expression modules further indicated a correlation between vaccine treatment and genes related to ER stress and unfolded protein response. Surprisingly, adjuvant-alone treatment had little impact on the spleen transcriptome. Additionally, the role of alternative splicing in the immune response was explored. We identified isoform switches in genes associated with immune regulation and inflammation, potentially influencing protein function. In conclusion, this study provides valuable insights into the transcriptomic changes in sheep spleen following vaccination with aluminum adjuvanted vaccines and aluminum hydroxide alone. These findings shed light on the molecular mechanisms underlying vaccine-induced immune responses and emphasize the significance of antigenic components in aluminum adjuvant mechanism of action. Furthermore, the analysis of alternative splicing revealed an additional layer of complexity in the immune response to vaccination in a livestock species.

## 1 Introduction

Among the main forms of aluminum used in vaccines as adjuvants, aluminum hydroxide is the most common (1). Aluminum salts are weak compared with other adjuvants and they are poor adjuvants for cell mediated immunity induction, but they have shown over the years to have a good safety record (2). Aluminum has also been shown to elicit a strong immune response (3) which can be Th1 or Th2 depending on the administration route (4). Rarely, adverse effects have been reported, such as increased IgE production and allergenicity; reduced renal function related aluminum accumulation, affecting brain and bone tissues or induction of autoimmune reactions (reviewed in 5). However, it is not clear that the cause of these effects is aluminum hydroxide itself (6).

Most of the studies performed to elucidate the mechanism of action of aluminum hydroxide-based adjuvants have been done *in vitro* or have focused on limited factors. The combinatory effect of all factors from the systems biology approach remains to be fully studied (4). The selective expression of specific gene sets modulates all the immune responses (7). Moreover, the identification of gene signatures has helped in the elucidation and enhancement of immune responses elicited by vaccines, for example the yellow fever vaccine. Therefore, the transcriptomic analysis of biological systems under the effects of aluminum hydroxide-based adjuvants could be an important approach to determine their molecular mechanisms (4).

In the framework of a collaborative project including veterinary pathologists, immunologists and genetics experts, a long-term experiment that aimed to analyze the effect of repetitive vaccination *in vivo* in sheep was performed. Animals were repetitively vaccinated with aluminum hydroxide containing vaccines or aluminum hydroxide alone. Subsequently, our research group studied the transcriptomic effect of repetitive vaccination and aluminum hydroxide administration in two tissues: PBMCs and encephalon (8, 9). In PBMCs both vaccine and adjuvant treatments stimulated the immune system. Though, in encephalon, the response was very mild, and a greater gene alteration was observed in sheep treated only with aluminum hydroxide. We also analyzed the differentially expressed lncRNAs in both tissues (9, 10), and it was observed that many immune-related lncRNAs were deregulated in PBMC after repetitive vaccination.

As mentioned above, the aim of vaccination is to generate an adaptive immune response against a given antigen. The development of the adaptive immune response occurs mainly in secondary lymphoid organs (7). Therefore, the study of the effect of vaccines and aluminum hydroxide in these secondary lymphoid organs could illuminate the mechanism of action of aluminum-based adjuvants and the elicited immune response. Moreover, the recent publication of a new and improved sheep genome and annotation, assembled using long-read sequencing data, allows a more accurate mapping and optimized functional profiling in RNA-seq studies (11).

The main aim of this work was to compare the transcriptomic profiles of repetitive administration of aluminum adjuvanted commercial vaccines and aluminum hydroxide alone in the ovine spleen.

To that end, we set out to unravel which are the differentially expressed genes and lncRNAs and enriched processes and pathways involved in the sheep spleen after repetitive administration of aluminum adjuvanted vaccines or aluminum hydroxide alone. Moreover, a gene co-expression analysis was performed. Lastly, we extended our study to enable analysis of genome-wide changes in specific types of alternative splicing and functional consequences of the detected isoform switches were predicted.

## 2 Materials and methods

### 2.1 Animals and experimental design

The Ethical Committee of the University of Zaragoza approved all experimental procedures (ref: PI15/14), fulfilling the requirements of the Spanish Policy for Animal Protection (RED53/2013) and the European Union Directive 2010/63 on the protection of experimental animals. This work was included in a long-term experiment and followed the same experimental design as described in Varela-Martínez et al. (9). Briefly, 21 three-month-old Rasa Aragonesa purebred lambs were selected and divided into 3 groups of 7 lambs. One group received aluminum-based subcutaneous commercial vaccines (the vaccine group). Another group (the adjuvant group) received equivalent doses of aluminum hydroxide (Alhydrogel, CZ Veterinaria, Spain). The control group only received phosphate-buffered saline (PBS). A total of 19 inoculations were administered in each group during a period of 475 days (from February 2015 to June 2016), following the recommended vaccination schedule.

### 2.2 Tissue collection and RNA extraction

Tissues for pathologic and transcriptomics studies were collected at necropsy. Samples of 1 g of spleen from each sheep were taken for RNA extraction and preserved in RNAlater solution (Ambion, Austin, TX, USA) at -80 °C. The experimental procedure to obtain RNA was similar to the one previously performed in other tissues from the same animals (9). Briefly, total RNA was isolated from spleen tissue using TRIzol reagent (Invitrogen, Carlsbad, CA, USA) and PureLink RNA Mini Kit (Invitrogen). 60 mg spleen tissue were homogenized in 1 mil of TRIzol Reagent using PrecellysR24 homogenizer (Bertin Technologies, Montigny-le-Bretonneux, France) combined with 1.4 and 2.8 mm ceramic beadsmix lying tubes (Bertin Technologies). RNA isolation was performed following manufacturer instructions and RNA was suspended in RNAse free water and stored at -80°C. RNA quantity and purity were assessed with NanoDrop 1000 Spectrophotometer (Thermo Scientific Inc, Bremen, Germany). RNA integrity wa assessed on an Agilent 2100 Bioanalyzer with Agilent RNA 6000 Nanochips (Agilent Technologies, Santa Clara, CA, USA), which estimates the 28S/18S (ribosomic RNAs) ration and the RNA integrity number (RIN value). The samples presented an average RIN value of 8.32 and a 260/280 ratio >1,7.

### 2.3 Library preparation and RNA sequencing

Before the library preparation, the quality of the RNA in the samples was measured to check the RNA integrity number (RIN). 18 samples with a RIN greater than 7,5 were selected. The total RNA libraries were prepared at CIBIR (Centro de Investigación Biomédica de La Rioja, La Rioja, Spain) from 300 ng of total RNA from each sample. The libraries were prepared using the Illumina Stranded Total RNA Prep with Ribo-Zero Plus (Illumina), following the manufacturer’s instructions. Total RNA was sequenced on a NovaSeq 6000 System sequencer (Illumina) at SGIker (SGIker, Universidad del País Vasco/Euskal Herriko Unibertsitatea, Spain). Paired end reads of 100 bp were sequenced and a mean sequencing depth of 123 million was obtained.

### 2.4 Total RNA expression analysis

All samples followed the same bioinformatic pipeline, generated for this work and written in a shell language script (**Supplementary Figure S1**). The quality of the raw samples was evaluated with Fastqc v 0.11.5 (12) to set the most appropriate preprocessing criteria. Trimmomatic v 0.39 (13) was used to filter out low confidence sequences, small sequences (> 36 bp) and adapters (allowing up to two mismatches). To ensure the complete rRNA depletion, the Bbduk function from BBTools v 37.62 (14) was used, loading non-redundant ribosomal sequences from the SILVA database (https://www.arb-silva.de/) as reference. After the data preprocessing, the quality of samples was checked again to ensure that the trimming and filtering were adequate.

The main analyses were performed using the ARS-UI_Ramb_v2.0 genome (11) and the ARS-UI_Ramb_v2.0 annotation release 104 from RefSeq (https://www.ncbi.nlm.nih.gov/refseq/). Kallisto v 0.48 (15) was used for transcript level quantification and the transcript sequences for its index were extracted using gffread v 0.12.1 (16). For differential lncRNA expression analysis, transcripts were also quantified in parallel using the Oar_rambouillet_v1.0 sheep genome and annotation from Ensembl release 103 (https://www.ensembl.org/index.html). The Ensembl annotation was extended using the unannotated lncRNAs identified in a study by our laboratory group (17).

The estimated abundance files of Kallisto were imported to R studio using the Tximport R package v 1.22.0 (18) and were normalized to transcript per million (TPM) to filter out all the genes with less expression than 1 TPM in more than half of the samples. Remaining ribosomal genes were also removed. The estimated gene counts were normalized using the median of ratios method from DESeq2 v 1.34.0 (19) and were ln-transformed (after adding 1 to all the expression values). To detect potential outliers or biases a principal component analysis (PCA) was performed, using the Factoextra R package v 1.0.7 (20). The Pearson correlation coefficient was used to analyze the correlation between samples within one experimental group and between experimental groups. The correlation between samples was visualized with a heatmap using the Pheatmap R package v 1.0.12 (21).

### 2.5 Differential expression and enrichment analysis

The differential expression analysis was performed using DESeq2. The 3 experimental groups were tested against each other, so 3 different comparisons were made: Vaccine vs Control (VvsC); Adjuvant vs Control (AvsC) and Vaccine vs Adjuvant (VvsA). The genes with a false discovery rate less than 0.05 after the Benjamini-Hochberg multiple testing correction and an absolute log2 fold change greater than 0.58 were selected as DEGs. Results were visualized using volcano and MA plots. The DEGs selected from the gene expression obtained using the Ensembl annotation were used to selectively explore differentially expressed lncRNAs (both annotated and unannotated lncRNAs).

The gene enrichment analyses were performed using the gProfileR2 R package v 0.2.1 (22) using Gene Ontology (GO) and Reactome databases. Human orthologs were used in the enrichment analyses and in the case of genes that mapped to many orthologs, one of them was selected. The statistical domain scope was set to all the filtered expressed genes and the enriched biological terms and pathways with an FDR (false discovery rate) adjusted p-value lower than 0.05 were selected.

For the differentially expressed lncRNAs obtained using the Ensembl annotation, correlations among expression levels of the lncRNAs and the closest annotated protein-coding gene (PCG) in a 100 Kb window were calculated. Statistical significances of the correlations were achieved with a test for association between paired samples (cor.test function in R) and a multiple testing correction method was applied with the false discovery rate (FDR) method (23). Pairs with an absolute Spearman correlation higher or equal than 0.6 and an FDR less than 0.05 were selected. The significant pairs may be potential candidates of lncRNAs that may act in cis regulation.

### 2.6 Gene co-expression network analysis

The co-expression analysis was performed using GWENA R package v 1.4.0 (24). The expression matrix normalized with the DESeq2’s median of ratios method was filtered to remove low variable genes and the 70% most variable genes were kept. The spearman correlation method was applied to construct the network. GWENA calculated the power and soft threshold to identify modules of co-expressed genes. The association of the eigengene (the principal component) of each module with the vaccine and adjuvant treatments was also analyzed using the Pearson correlation method with a p-value threshold <0.05.

We performed gene enrichment analyses with the genes of each module using gProfileR2 and the terms with an FDR less than 0.05 were selected. We set the statistical domain scope of the enrichment analyses to all the genes that were kept after the low variable gene filtering. Finally, the co-expression module correlations were visualized using Cytoscape v 3.7.1 (25). In addition to the enrichment analysis, the hub genes of treatment associated modules were obtained. For that purpose, module membership (correlation of gene and eigengene expression values) and gene significance (correlation of gene expression and treatment parameter) values were calculated. Hub genes were defined as those among the ≥ 85th percentile for both values. Those genes are likely “key drivers” and might play important roles in the treatment.

### 2.7 Differential transcript usage analysis

The differential transcript usage (DTU) analysis was performed with IsoformSwitchAnalyzeR R package v 1.16.0 (26). Transcript level abundance estimates were imported to R and the gene list was reduced by removing single-isoform genes, genes lowly expressed (<1TPM) and transcripts with low isoform fraction (IF<0.01). The DTU statistical test was performed with DEXSeq (27), which is implemented in IsoformSwitchAnalyzeR, comparing the 3 experimental groups against each other (Vaccine vs Adjuvant, Vaccine vs Control and Adjuvant vs Control). To determine significant isoform switches we applied a differential isoform fraction (dIF) threshold of 0.1, an FDR threshold of 0.05 and required to be another isoform with the opposite effect size. In order to predict functional consequences of the isoform switches with IsoformSwitchAnalyzeR we analyzed the nucleotide sequences and protein sequences with CPC2 (28) to assess protein coding capability, Pfam (29) to find protein domains, signalP 5.0 (30) to find signal peptides and IUPred2A (31) to find intrinsically disordered protein regions.

## 3 Results

### 3.1 Dataset exploratory analysis

18 libraries with a RIN score greater than 7.5 were sequenced (6 samples per group) and a mean of 118.13 ± 4.5 million reads per library were generated (**Supplementary Table S1**). After filtering out low-quality reads, ribosomal sequences and adapters, a mean of 111.24 ± 4.6 million reads (94%) per library remained. The pre-processed samples were pseudo-aligned with Kallisto and a mean of 43.41 ± 1.47% of the reads were pseudo-aligned against the RefSeq transcriptome and 33.90 ± 1.18% against the Ensembl extended transcriptome.

In the first exploratory analysis using PCA, samples 135-B and 127-B were considered technical outliers and were excluded from further analyses. These outliers could also be detected at the sequencing quality control step. After excluding these samples, 24866 expressed genes were identified using the RefSeq annotation. 12 annotated rRNA genes were still found and removed. 17385 non-rRNA genes with more expression than 1 TPM in more than half of the samples were selected for further analysis. The PCA after outlier removal showed an overlap between the Control and Adjuvant groups but a clear separation of Vaccine samples from the rest of the samples with the first two principal components (**Supplementary Figure S2**).

### 3.2 Differential expression analysis

**Figure 1:**
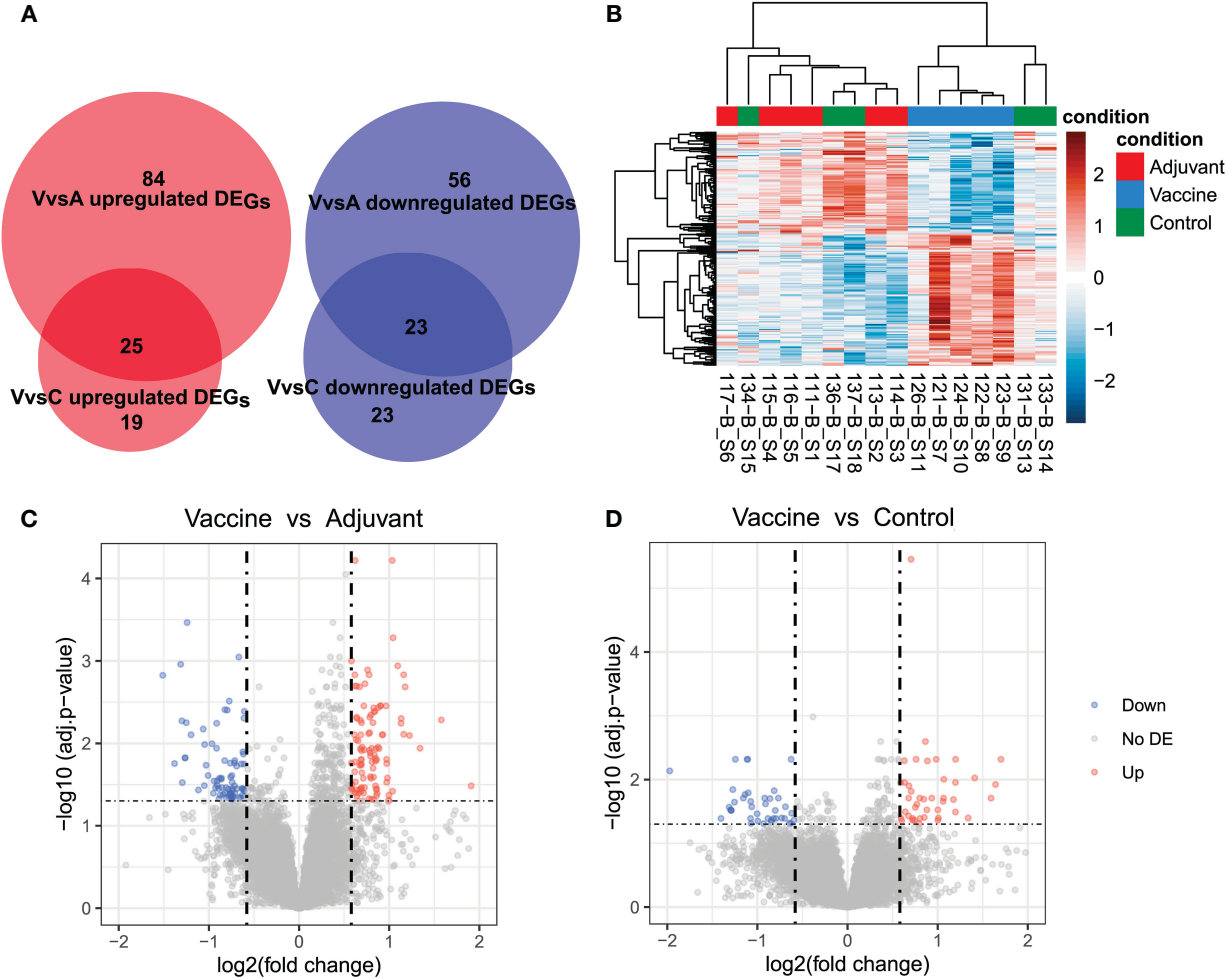
Differential expression analysis results. **(A)** Venn diagram that shows the number of upregulated and downregulated genes found in the comparisons of Vaccine vs Adjuvant (VvsA) and Vaccine vs Control (VvsC). **(B)** Heat map that shows the normalized (with DESeq2’s median of ratios) and ln transformed expression of differentially expressed genes (DEG) detected in all the comparisons. The high expression values were plotted in red and low values in blue. **(C, D)** Volcano plots of the differentially expressed genes (DEG) in the Vaccine vs Adjuvant **(C)** and Vaccine vs Control **(D)** comparisons. Downregulated genes (down) are plotted in blue and upregulated genes (up) in red. The genes that were not differentially expressed (no DE) are plotted in gray.

The VvsA comparison showed the highest number of differentially expressed genes, with 188 genes, of which 109 displayed an increased expression in Vaccine samples and 79 a decreased expression (**Figure 1A**). In the VvsC comparison 90 DEGs were identified, of which 44 DEGs were upregulated and 46 downregulated, and only 2 DEGs were identified in the AvsC comparison (all of them were downregulated). Twice as many DEGs were identified in VvsA comparison than in VvsC comparison and 48 DEGs were shared between both comparisons (23 downregulated DEGs and 25 upregulated DEGs) (**Figure 1A**). We analyzed the correlation between samples only using the DEGs detected in the three comparisons, and we distinguished two main sample clusters: one mostly composed of Vaccine samples and another with Control and Adjuvant samples (**Figure 1B**).

FDR values were greater in the VvsA comparison than in the VvsC comparison (**Figures 1C, D**). The detected DEGs mostly did not display severe deregulation. However, we can observe that certain genes appear highly deregulated. In the VvsA comparison FTL (L2FC: 8.57; FDR: 8.94E-05), LOC114114660, U1 spliceosomal RNA (L2FC: -9.36; FDR: 4.2E-02) and LOC114113983, ortholog of RPS23 (L2FC: -5.04; FDR: 2.34E-02); were highly deregulated. The LOC114114660 (L2FC: -11.09; FDR: 2.99E-02) and LOC114113983 (L2FC: -5.33; FDR: 3.72E-02) genes also appeared highly deregulated in VvsC comparison. In the AvsC comparison all the genes were very deregulated: FTL (L2FC: -6.87; FDR: 0.04) and LOC101122601 (L2FC: -6.15; FDR: 2.38E-03).

The 10 most significant DEGs of each comparison are shown in **Table 1**. The AvsC comparison is not shown in the Table because only 2 DEGs were identified. The gene with a lower FDR value in the VvsA comparison, SELENOK, is involved in endoplasmic reticulum (ER) stress responses and immune responses. Among the total DEG list of the VvsA comparison, some immune-related genes (SERPINB1, MZB1 or S100A9), cell cycle-related genes (CDCA8 or E2F2), kinesin family members (KIF11, KIF22, KIFC1, KIF4A and KIF2C) and 32 histone genes were detected. In the VvsC comparison, DEGs associated with similar processes or structures to those identified in the VvsA comparison (immune response and cell cycle) were detected. The complete list of DEGs is shown in **Supplementary Table S2**.

**Table 1:** The 10 first detected differentially expressed genes (DEG), ranked by adjusted p-value, in Vaccine vs Adjuvant and Vaccine vs Control comparisons. The Adjuvant vs Control comparison is not shown because only 3 DEGs were identified. The table also shows the log2 fold-change of each gene.

Upregulated gene name	log_2_ fold-change	Adjusted p-value	Downregulated gene name	log_2_ fold-change	Adjusted p-value
Top 10 DEGs in Vaccine vs Adjuvant comparison					
SELENOK	0.62	6.05E-05	FAM181A	-1.24	3.42E-04
SERPINB1	1.03	6.05E-05	LOC121817230	-0.67	8.98E-04
FTL	8.57	8.94E-05	LOC121817744	-1.31	1.10E-03
CDCA8	1.04	5.24E-04	LOC121820202	-1.51	1.50E-03
HSP90B1	0.58	1.00E-03	TMEM44	-0.77	3.08E-03
LOC11410968	1.09	1.15E-03	RYR1	-0.82	3.91E-03
NUF2	0.76	1.29E-03	LOC121818741	-0.80	3.96E-03
CCNF	0.62	1.47E-03	C17H4orf33	-0.61	4.10E-03
LOC10560297	0.78	1.47E-03	COL4A5	-0.61	4.95E-03
LOC10699041	1.16	1.47E-03	PARP3	-1.30	5.35E-03
Top 10 DEGs in Vaccine vs Control comparison					
SELENOK	0.71	3.53E-06	FAM181A	-1.11	4.81E-03
LOC10112096	0.86	2.53E-03	JUN	-0.63	4.81E-03
DEPP1	0.98	4.81E-03	LOC101122601	-5.69	4.81E-03
E2F2	0.76	4.81E-03	LOC121818087	-1.11	4.81E-03
LOC10110377	1.70	4.81E-03	NTSR1	-1.24	4.81E-03
LOC10699041	1.20	4.81E-03	LOC114117120	-1.97	7.32E-03
CRELD2	0.63	5.06E-03	LOC121816775	-1.27	1.44E-02
LOC10560281	0.89	5.10E-03	MYO6	-0.81	1.50E-02
S100A9	1.41	9.46E-03	LOC121817866	-1.10	1.61E-02
LOC10111479	1.07	9.92E-03	LOC121817744	-1.14	1.72E-02

#### 3.2.1 Expression of annotated and unannotated lncRNAs

**Figure 2:**
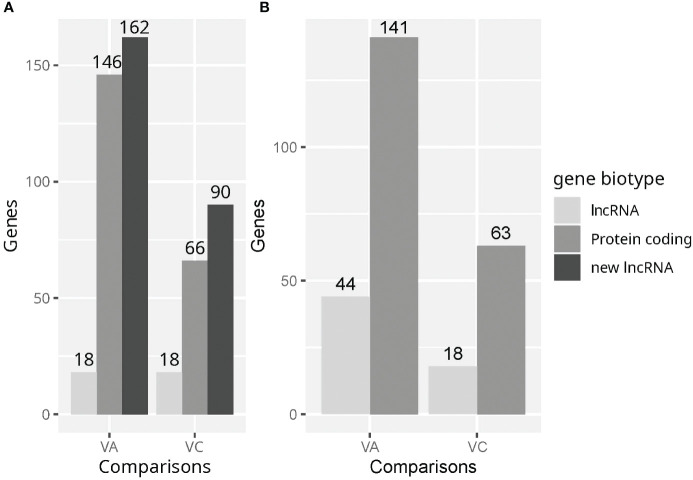
The amount of differentially expressed protein coding genes and lncRNAs in the Vaccine vs Adjuvant (VvsA) and Vaccine vs Control (VvsC) comparisons. **(A)** Differentially expressed genes using the Ensembl annotation supplemented with unannotated lncRNAs. **(B)** Differentially expressed genes using the RefSeq annotation. Other RNA types (such as snoRNAs, snRNAs, pseudogenes or IG_V_genes) adding up to very few genes were not considered.

We analyzed lncRNA and protein coding gene detection and differential expression using two annotations (Refseq and an extended Ensembl annotation). This would serve to check the completeness of these annotations in terms of lncRNA genes and to validate in an independent dataset the set of unannotated lncRNAs from a previous study (17). In samples quantified using the Ensembl extended annotation 21688 genes remained after the minimum expression filter and ribosomal gene filters. Of those, 1400 genes were already annotated lncRNAs and 5990 were unannotated lncRNAs. In addition, 146 unannotated lncRNAs were found dysregulated in the VvsA comparison and 66 in the VvsC comparison (**Figure 2**) Using the Ensembl extended annotation 326 DEGs were identified in the VvsA comparison, 174in the VvsC comparison and no DEG was detected in the AvsC comparison. The number of detected protein coding genes in both comparisons were very similar using both annotations (**Figure 2**). The amounts of already annotated lncRNAs were also similar, except in the VvsA comparisons, where twice as many lncRNAs were detected using the RefSeq annotation. On the other hand, the addition of unannotated lncRNAs increased total DEG amount by two-fold in the VvsA and VvsC comparisons using the Ensembl annotation.

The VvsA comparison showed 19 significant lncRNA-PCG correlations among the differentially expressed lncRNAs, while the VvsC comparison showed 7 significant pairs (**Supplementary Table S3**). All significant correlations were negative and both comparisons shared 5 significant lncRNA-PCG pairs: MSTRG.6683- FNDC3A (ρ=-0.83, FDR= 1.81E-03; intronic antisense lncRNAs), MSTRG.22630- IDH3A (ρ=-0.79, FDR= 1.81E-03; intergenic lncRNA), MSTRG.53494- RAB8B (ρ=-0.72, FDR= 1.81E-03; intronic antisense lncRNA), MSTRG.2195- SEC22B (ρ=-0.63, FDR= 1.81E-03; antisense lncRNA) and MSTRG.16999- YAP1 (ρ=-0.64, FDR= 1.81E-03; intergenic lncRNA). There were genes related to the vesicle transport machinery (RAB8B and SEC22B), RNA binding activity (FNDC3A and EIF4B) and cell growth (YAP1, ABI1, DLG1 and PPP2R5C), among others.

### 3.3 Functional profiling of the differentially expressed genes

Functional profiling analyses were performed using the DEGs from the comparisons VvsA and VvsC. Because of using the human orthologs of the annotated sheep genes the final DEG lists were reduced to 97 and 45 DEGs in VvsA and VvsC comparisons, respectively. The statistical domain scope for the enrichment tests was also reduced to 12541 genes.

**Figure 3:**
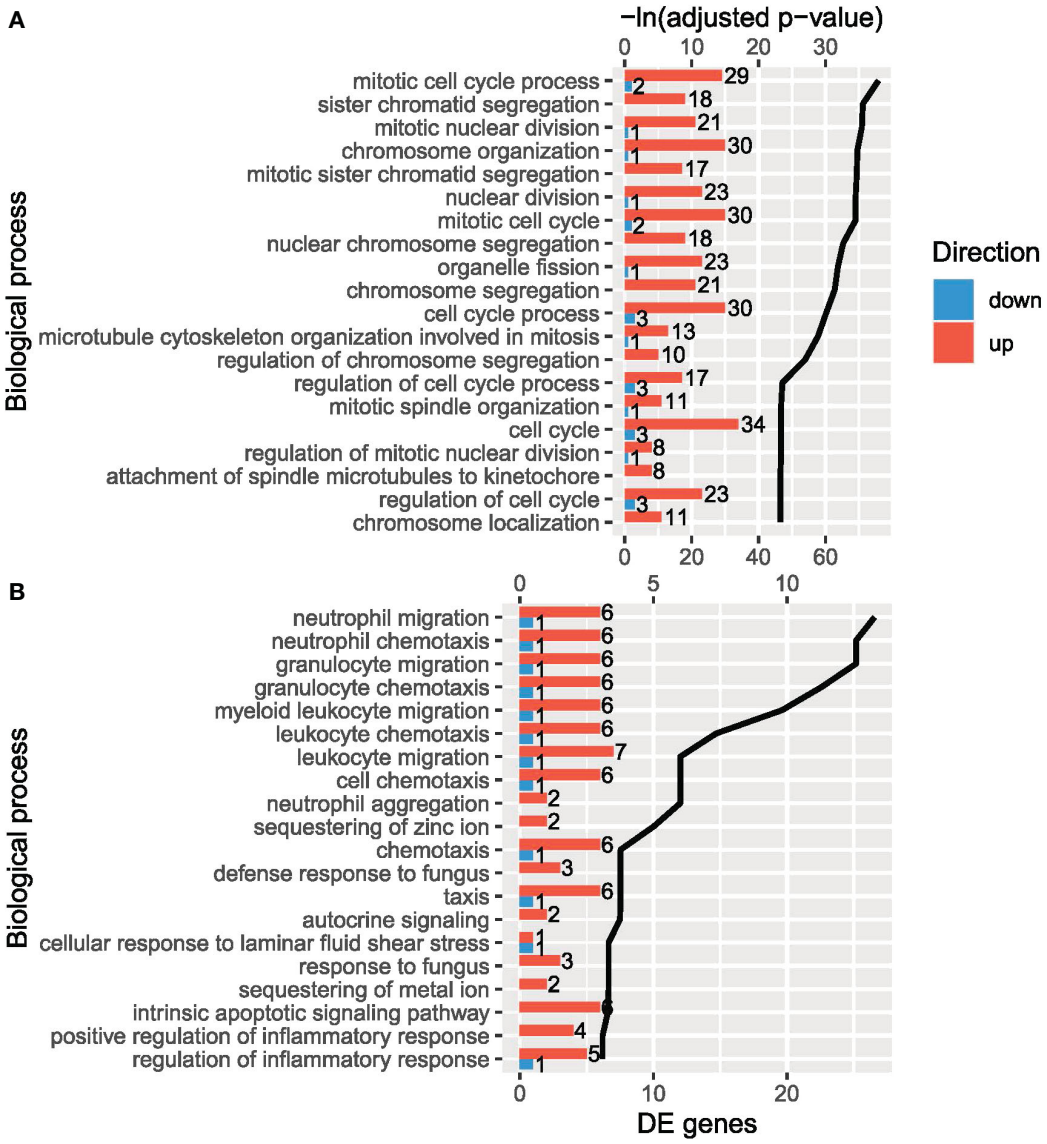
The most significant enriched biological processes from Gene Ontology (ranked by adjusted p-value) in the human orthologs of the differentially expressed genes (DEGs) in **(A)** VvsA comparison and **(B)** VvsC comparison. The bars show the amount of upregulated (up) and downregulated (down) DEGs and the black line shows the -ln adjusted p-value of each term.

In the VvsA comparison, 179 biological processes from GO were enriched. Several cell cycle processes were among the most significantly enriched terms (**Figure 3A**, **Supplementary Table S4A**), for instance: chromosome organization (FDR: 8.20E-16), nuclear division (FDR: 1.05E-15), cell cycle (FDR: 7.16E-11) or mitotic cell cycle (FDR: 1.05E-15). Cell cycle-related processes involved E2F2, CDK1, some Kinesin family members (members 11, 22, 2C, 4A or C1) and many histone genes, among others. In addition to cell cycle terms, there were other terms such as response to endoplasmic reticulum stress (FDR: 7.21E-03), response to topologically incorrect protein (FDR: 1.44E-02), response to unfolded protein (FDR: 3.78E-02) and positive regulation of interleukin-6 production (FDR: 3.79E-02). The overrepresentation of Reactome pathways revealed many immune-related pathways, such as, HCMV Late Events (FDR: 1.64E-03), Immune System (FDR: 2.09E-02) or Adaptive Immune System (FDR: 3.09E-02). Genes such as C3, S100A9, ISG20, HSP90B1, PADI2, AGER, BIRC5, CCNF, COL17A1 and FTL were involved in these pathways.

In the VvsC comparison, 20 biological processes from GO were enriched (**Figure 3B**, **Supplementary Table S4B**). Some immune-related processes were the most significantly enriched terms, specifically related to the migration/chemotaxis of neutrophils and granulocytes, with the same 7 DEGs involved in these processes: EDN3, LGALS3, TNFAIP6, TREM1, S100A12, S100A8 and S100A9.

### 3.4 Gene co-expression analysis

**Figure 4:**
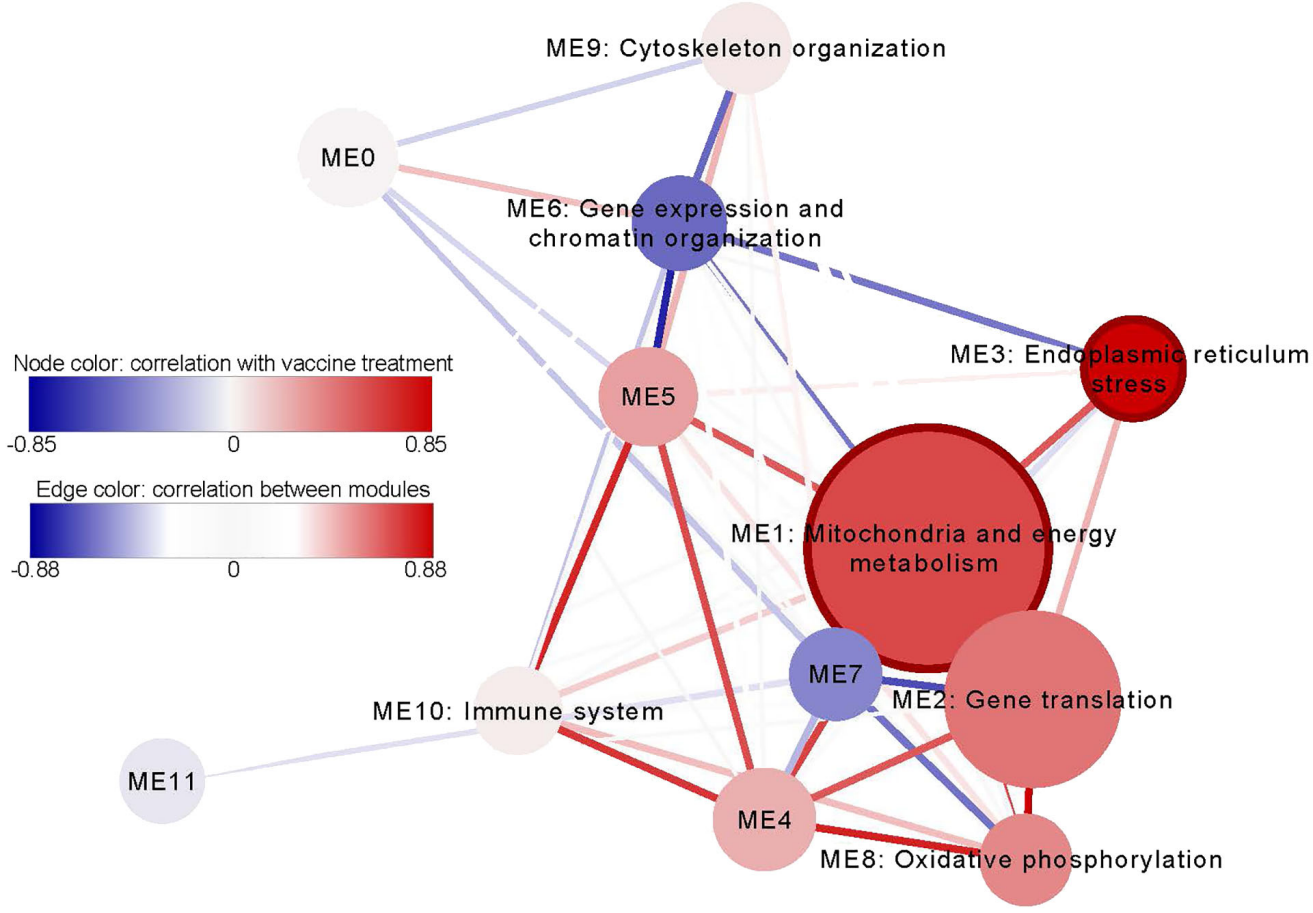
The network of co-expressed gene modules detected in all samples. The node size represents the amount of genes of the node and the node color visualizes the correlation of the module with the vaccine treatment. The modules that are significantly correlated (p-value < 0.05) with vaccine treatment have a bold edge. The color of the lines that connect the modules together (edges) visualizes the correlation between module eigengenes and their thickness correspond the p-value of this correlation. The modules that have few and/or heterogeneous GO and Reactome enrichment terms were not named.

We used the GWENA program to detect co-expressed gene modules and construct a gene co-expression network. 12169 genes remained after filtering out genes with low variability. 12 modules of co-expressed genes that ranged from 217 to 4670 genes were detected (**Figure 4**, **Supplementary Table S5A**). We analyzed the correlation between the eigengene of each module and vaccine and adjuvant alone treatments. We detected 2 modules significantly correlated with the vaccine treatment: ME1 (R: 0.6, p-value: 1.47E-02) and ME3 (R: 0.85, p-value: 3.54E-05). No module was found to be significantly correlated with the adjuvant treatment.

The co-expression modules were functionally characterized by gene set enrichment of overrepresented GO and Reactome terms (**Supplementary Table S5B**). The module ME3, which showed a higher significant correlation with the vaccine treatment, was enriched for several ER stress-related GO terms, such as, response to endoplasmic reticulum stress (FDR: 4.86E-04), response to topologically incorrect protein (FDR: 1.21E-03), response to unfolded protein (FDR: 2.88E-033), ERAD pathway (FDR: 2.46E-02) or endoplasmic reticulum unfolded protein response (FDR: 2.47E-02). Module ME1 had various enriched terms associated with mitochondria and energy metabolism, for instance, mitochondria envelope (FDR: 5.3E-03), mitochondria membrane (FDR: 2.70E-02), Respiratory electron transport, ATP synthesis by chemosmotic coupling, and heat production by uncoupling proteins (FDR: 5.37E-03) or respiratory electron transport (FDR: 3.46E-02). We also detected a module related to the immune system (ME10) which includes enriched GO terms such as immune system processes (FDR: 1.61E-02) or immune response (FDR: 2.09E-02).

The hub genes of the treatment related modules were checked (**Supplementary Table S5C**). Module ME1 had 317 genes classified as hub genes, of which 2 were differentially expressed, and the module ME3 had 93 genes, of which 26 were differentially expressed. The hub genes of the ME3 module were enriched for several ER-related terms, such as, protein localization to endoplasmic reticulum (FDR: 8.72E-08), response to unfolded protein (FDR: 1.35373E-07), response to endoplasmic reticulum stress (FDR: 1.82E-06), SRP-dependent cotranslational protein targeting to membrane (FDR: 1.99E-06), endoplasmic reticulum to cytosol transport (FDR: 1.25E-05) and ubiquitin-dependent ERAD pathway (FDR: 1.73E-05), in addition to some glycosylation related terms such as protein N-linked glycosylation (FDR: 9.19E-06), glycoprotein metabolic process (FDR: 0.00055) and glycoprotein biosynthetic process (FDR: 0.0069). In contrast, hub genes from ME1 module were enriched for energy metabolism-related terms, such as, aerobic respiration (FDR: 4.68E-07), tricarboxylic acid cycle (FDR: 9.13E-05), oxidative phosphorylation (FDR: 0.0012), proton motive force-driven ATP synthesis (FDR: 0.0026) and ATP biosynthetic process (FDR: 0.0098). See a more detailed list of enriched terms in the ME1 and ME3 module in **Supplementary Tables S6A** and **S6B**, respectively.

### 3.5 Identification of isoform switches and alternative splicing events

**Figure 5:**
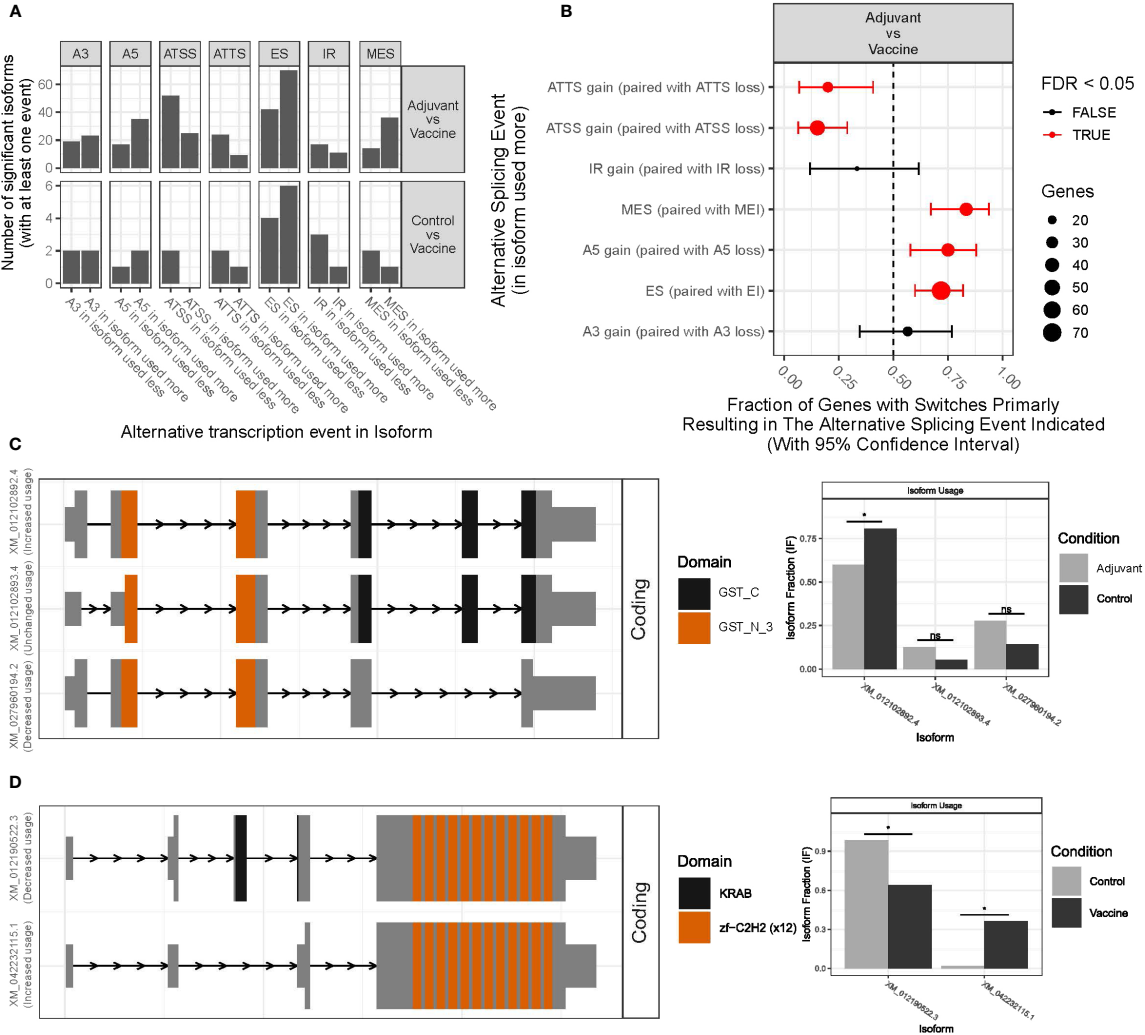
Differential transcript usage (DTU) analysis results. **(A)** The number of significant isoforms that have gained or lost alternative splicing sites in each comparison between experimental groups (VvsA and VvsC). **(B)** Enrichment of specific splice events in the VvsA comparison. The red colored gene fractions indicate that FDR<0.05. **(C)** DTU results for TLR10 gene. **(D)** DTU results for CST7 gene. * means p-value < 0.05; ns means no significant differences between the two conditions.

We used transcript level expression to identify patterns of isoform usage and alternative splicing between different treatments, with a special focus on the transcript-level changes upon immune activation induced by the vaccine treatment. Performing this analysis was possible because of the very high sequencing depth in this RNA-seq study. In total, 332 significant DTU events were detected in 267 genes with dIF higher than 0.1. The isoform switches were categorized according to the type of alternative splicing event happening between transcripts. Among the significant DTU events, single exon skipping (ES) and alternative transcription start sites (ATSS) were the most common (**Figures 5A, B**). Other detected categories include Alternative 5’ Donor Sites, multiple exons skipping or alternative transcription termination sites (ATTS). Moreover, 62 of them were determined as switching genes by IsoformSwitchAnalyzeR program, which classifies a gene as switching when it has large opposite changes in isoform usage across conditions and at least one of the changes is used in a statistically significant way. Many genes without differential gene expression showed significant isoform changes. Among the 3 comparisons between treatments, three switches with consequences were detected in the AvsC comparison, and 5 in the VvsC comparison while 52 were detected in the VvsA. In this comparison, most of the isoforms showed a protein domain gain, a loss of intron retention or a gain of intrinsically disordered regions (IDR). In theAvsC comparison genes with different isoforms included CCDC116, GSTO1_like and TMEM214. In the VvsC comparison, switched genes were EIF2Ak4, MYADM-like, PLAG1, ZNF112 and TNNT3, which are mainly involved in transcription and protein synthesis regulation. None of these switched genes presented differential expression.

Among the switched genes, some are worth mentioning. For example, GST proteins are involved in the metabolism of carcinogens and xenobiotics, namely chemicals extrinsic to the normal metabolism of the organism. It has been seen that the use of the 3 different isoforms of the GST-like gene (LOC101116273) is quite different in animals with aluminum-based adjuvant treatment and controls (**Figure 5C**). On the other hand, ZNF112 gene codifies a protein which is predicted to be involved in regulation of transcription by RNA Polymerase II. In this work it has been detected that in the ZNF112 gene two isoforms were statistically differently used in controls and vaccinated animals; the isoform mainly used in control animals has a KRAB domain, a repressor of transcription, whis is absent in the isoform mainly used in vaccinated animals. This switch could indicate a different regulation of transcription in vaccinated animals (**Figure 5D**).

Among the genes with consequences in the VvsA comparison, many genes associated with the immune system were detected, namely TLR10, CST7, EPSTI1, GON4L or HNRNPLL. CST7 gene for example, may play a role in immune regulation through the inhibition of a unique target in the hematopoietic system. Although CST7 was not detected in the differential expression analysis, two different isoforms were detected for CST7 and the isoform with an increased usage in the vaccine group had a signal peptide (**Supplementary Figure S3A**). On the other hand, TLR10, a member of the Toll-like receptor (TLR) family that has a crucial role in pathogen identification and activation of innate immunity, had also two annotated isoforms. As CST7, the one that had an increased usage in the vaccine group also gained a signal peptide compared to the less used isoform (**Supplementary Figure S3B**). Finally, some genes associated with the immune system were detected among the genes with switching isoforms but without predicted functional consequences, for example, GAPT or LOC105601870 (TIR domain-containing adapter molecule 2 in *Ovis aries*).

## 4 Discussion

Although aluminum hydroxide has been widely employed as a vaccine adjuvant over years, its mechanism of action remains unclear (5). The study of gene signatures has helped to predict and improve the immune effect of some vaccines (4). In the present work, the transcriptomic signature of the spleen from lambs that were injected with commercial vaccines (Vaccine group), aluminum hydroxide only (Adjuvant group) or PBS (Control group), has been analyzed using RNA-seq to elucidate the effect of the aluminum hydroxide after repetitive vaccination *in vivo*. This work is, as far as we know, the first study that has analyzed the transcriptome of the sheep spleen after a repetitive vaccination experiment. The spleen, as the biggest secondary lymphoid organ in the human body, has been considered a potential organ for nanovaccines vaccination. Moreover, the depth achieved in the RNA sequencing has allowed the study of the immune response to vaccination at isoform level across all the analyzed conditions.

As previously stated in Varela-Martínez et al. (8), in the experimental design priority was given to the homogeneity of the individuals analyzed in different groups, so animals of the same herd without any vaccination before our experiment were included. A period of adaptation to the new experimental flock was kept, and they were in the best conditions of feeding and temperature, all of them controlled. On the other hand, it was a repetitive vaccination experiment, so it was very difficult to dissect the effect of each vaccine separately. It was expected to see the cumulative effect of all the inoculations, without ruling out that the latter had a greater effect on the response of the animals than the previous ones.

In this work, we used the newly improved genome sequence and annotation for sheep ARS-UI_Ramb_v2.0 (RefSeq annotation) and the previous Oar_rambouillet_v1.0 assembly with the Ensembl annotation extended with 12302 extra lncRNA genes (17) to quantify gene expression. ARS-UI_Ramb_v2.0 annotation had greater transcriptome pseudo-alignment rates, even after the inclusion of extra lncRNAs in the Oar_rambouillet_v1.0 annotation, which reflects its better quality. Nevertheless, the detection of the expression of about 50% of the added lncRNA genes in a single tissue validates the novel lncRNAs assembled in that previous study. Besides, the addition of these unannotated lncRNAs represented a more than five-fold increase in the amount of differentially expressed lncRNAs. In comparison with human and murine annotations, livestock genomes are under annotated in terms of lncRNA genes, including the latest sheep reference annotations (32). Therefore, the annotation and the understanding of how these genes are expressed in the genomes of farm animals is essential for bridging the genotype-phenotype gap (33), and for instance, identifying lncRNAs that are involved in immune responses to pathogens and vaccines.

One of the most surprising results in the comparisons performed in this work was the lack of differences between the adjuvanted animals and the controls (AvsC comparison). The exploratory analyses revealed a great extent of overlap of the Control and Adjuvant groups and only 2 genes were found to be differentially expressed when comparing these groups. On the contrary, Al hydroxide-containing commercial vaccine inoculations did lead to changes in gene expression compatible with an immune reaction to the vaccine components. These results seem to indicate that, without the presence of the antigen, aluminum or its effect does not reach the spleen, or it does so very poorly. (34) analyzed the aluminum content in sheep lymph nodes after being inoculated with aluminum-based commercial vaccines, only Al hydroxide or PBS in the same experiment. They observed that aluminum was translocated by macrophages from the injection site to lymph nodes in the group that was injected with commercial vaccines. Nonetheless, in the groups that were inoculated with aluminum hydroxide or PBS alone, the metal content of lymph nodes was almost the same. Since both lymph nodes and spleen are secondary lymphoid organs, a similar translocation pattern may be expected in the spleen, even if aluminum particles could also reach the spleen through the blood.

The 2 DEGs in the AvsC comparison (FTL and LOC101122601) could be related to the effect of Al adjuvants. FTL is a member of the ferritin complex involved in iron transport. Since pathogens need iron for growing, iron-withholding-related genes, such as FTL, are usually upregulated in response to infections (35). Moreover, a study carried out in rat brain showed that ferritin absorbed the aluminum *in vivo* and that ferritin may play an important role in the detoxification of aluminum in cells (36). Unexpectedly, while FTL was upregulated in Vaccine samples in comparison to Adjuvant samples, it was downregulated in the AvsC comparison. This could be linked with the loss of iron homeostasis after chronic aluminum intoxication that was observed in rat liver (37). Further research on the effect of aluminum on iron homeostasis and vesicle trafficking is needed to fully understand these results.

Regarding the commercial vaccines’ treatment, many DEGs were detected in both comparisons (90 DEGs in the VvsC comparison and 188 DEGs in the VvsA comparison). The enriched processes and pathways were mainly related to cell cycle and the immune system. The detection of cell cycle-related terms could be due to the clonal expansion that the lymphocytes undergo during the adaptive immune response (38). Regarding the immune-related genes, we found many similar genes between the VvsA and VvsC comparisons, e.g., S100A9, S100A12, LGALS3, TNFAIP6, TREM1 or EDN3. The processes and pathways enriched in the VvsA comparison were clearly related to an adaptive immune response. Overall, the enriched pathways and processes indicate that an immune response was happening in the sheep treated with commercial vaccines. In livestock, few studies have been made on the spleen transcriptome after an infection and the enriched terms in these works were similar to those detected in the present study (39–42).

The co-expression analysis performed with GWENA revealed similar results. Interestingly, two of the co-expression modules (ME1 and ME3) were correlated with the vaccine treatment. The enriched GO terms and Reactome pathways in the ME1 module were mainly related to mitochondria and energy metabolism. A tendency of aluminum hydroxide to increase basal mitochondrial metabolism, seemingly linked to energetic requests to support phagocytosis and cytokine production, has been observed in human PBMCs exposed to the adjuvant and whole vaccines (43). Moreover, the ME3 module was mainly enriched in terms related to ER stress and the unfolded protein response (UPR) signaling cascade. The response to ER stress is essential for resolving secretory stress and survival of highly secretory cells such as immunoglobulin producing plasma cells and other immune cells (44, 45). During ER stress, the accumulation of unfolded proteins leads to activation of the UPR cascade, of which IRE1alpha and XBP1 are part, to return the homeostasis of the ER (46). Multiple studies have reported that IRE1alpha-XBP1 signaling contributes to the innate immune responses triggered by various toll-like receptors (47). Recent studies have started to shed light on the pivotal relationship between the immune response and alternative splicing. The role of alternative splicing has been proposed to serve as a mechanism for genes to adapt to dynamic immune challenges such as infection (48). This includes changes such as exon skipping, and the utilization of different transcription start sites. Moreover, using information from predicted protein domains, sequence coding potential and other features it is possible to predict some functional consequences of a change in isoform usage. In this work, these analyses are limited by the sheep genome annotation and structural and functional information in databases about the analyzed genes, since other changes not included in these predictions could also have a functional impact.

Because alternative splicing plays this crucial role in the immune response, it is worth analyzing in relation to immune response in vaccination but understanding the use of different isoforms in different treatment groups is not straightforward. One of the clearest examples is the ZNF112 zinc finger protein coding gene. In this gene, the isoform mainly used in the control animals has a KRAB domain which is absent in the isoform mainly used in vaccinated animals. This switch could indicate a different regulation of transcription in vaccinated animals. In a different scenario, a similar process was described by Vitting-Seerup and Sandelin (49). An isoform switch in the zinc finger protein, ZNF12, resulted in a loss of the transcriptional inhibitory KRAB domain. This isoform switch was associated with worse survival rates in 5 cancer types.

Thus, isoform diversity and the consequent impact on protein domain expression reveal an additional layer of complexity during immune response and by extension, to vaccine antigens. In this work, to shed light on the mechanism of action of aluminum-adjuvanted vaccines, we have profiled the transcriptomic responses after repetitive vaccination with aluminum adjuvanted vaccines and aluminum hydroxide alone in sheep. We show that, in spleen, contrary to what was previously observed in blood cells, aluminum hydroxide alone does not elicit any transcriptomic alteration, which suggests that the antigen component is necessary for its transport to the spleen. In animals treated with complete vaccines there were differentially expressed coding genes related to an immune response and a comparable amount of differentially expressed lncRNAs. Besides, isoform-level analyses revealed changes in alternative splicing patterns due to the experimental treatments and independent from differential gene expression. This process seems to diversify the function of the immune response to vaccination.

## Data availability statement

The datasets presented in this study can be found in online repositories. The names of the repository/repositories and accession number(s) can be found below: https://www.ncbi.nlm.nih.gov/geo/query/acc.cgi?acc=GSE, GSE247587.

## Ethics statement

All procedures were carried out under Project License PI15/14 approved by the Ethics Committee for Animal Experiments of the University of Zaragoza. The care and use of animals were performed according to the Spanish Policy for Animal Protection RD53/2013, which meets the European Union Directive 2010/63 on the protection of animals used for scientific purposes.

## Author contributions

AG-S: Formal analysis, Investigation, Methodology, Visualization, Writing – original draft, Writing – review & editing. MB-A: Formal analysis, Investigation, Methodology, Visualization, Writing – original draft, Writing – review & editing. EV-M: Methodology, Visualization, Writing – original draft, Writing – review & editing. NA: Investigation, Methodology, Writing – review & editing. MP: Methodology, Resources, Writing – review & editing. LL: Conceptualization, Funding acquisition, Methodology, Resources, Writing – review & editing. BJ: Conceptualization, Funding acquisition, Project administration, Supervision, Visualization, Writing – original draft, Writing – review & editing.
