# Supplementary material for: Identifying transcriptomic profiles in ovine spleen after repetitive vaccination
Source: Front Immunol. 2024 Jul 12;15:1386590. doi: 10.3389/fimmu.2024.1386590 (PMC11284609; doi:10.3389/fimmu.2024.1386590)
Supplement: Supplementary file 1 [file DataSheet_1.pdf]

Supplementary Material

Transcriptomic signature after repetitive vaccination in ovine spleen

Guisasola-Serrano A[1], Bilbao-Arribas M[1,3], Varela-Martínez E[1], Abendaño N[1], Pérez M[2], Luján L[2], Jugo BM[1]

[1]Genetics, Physical Anthropology and Animal Physiology Dpt., Faculty of Science and Technology, University of the Basque Country (UPV/EHU), 48940 Leioa, Spain

[2]Animal Pathology Dpt., Faculty of Veterinary, University of Zaragoza, Zaragoza, Spain

[3]Present Address: CIMA, Universidad de Navarra, Pamplona, Spain*

Correspondence: Corresponding Author: begonamarina.jugo@ehu.eus

1 Supplementary Figures and Tables

1.1 Supplementary Figures

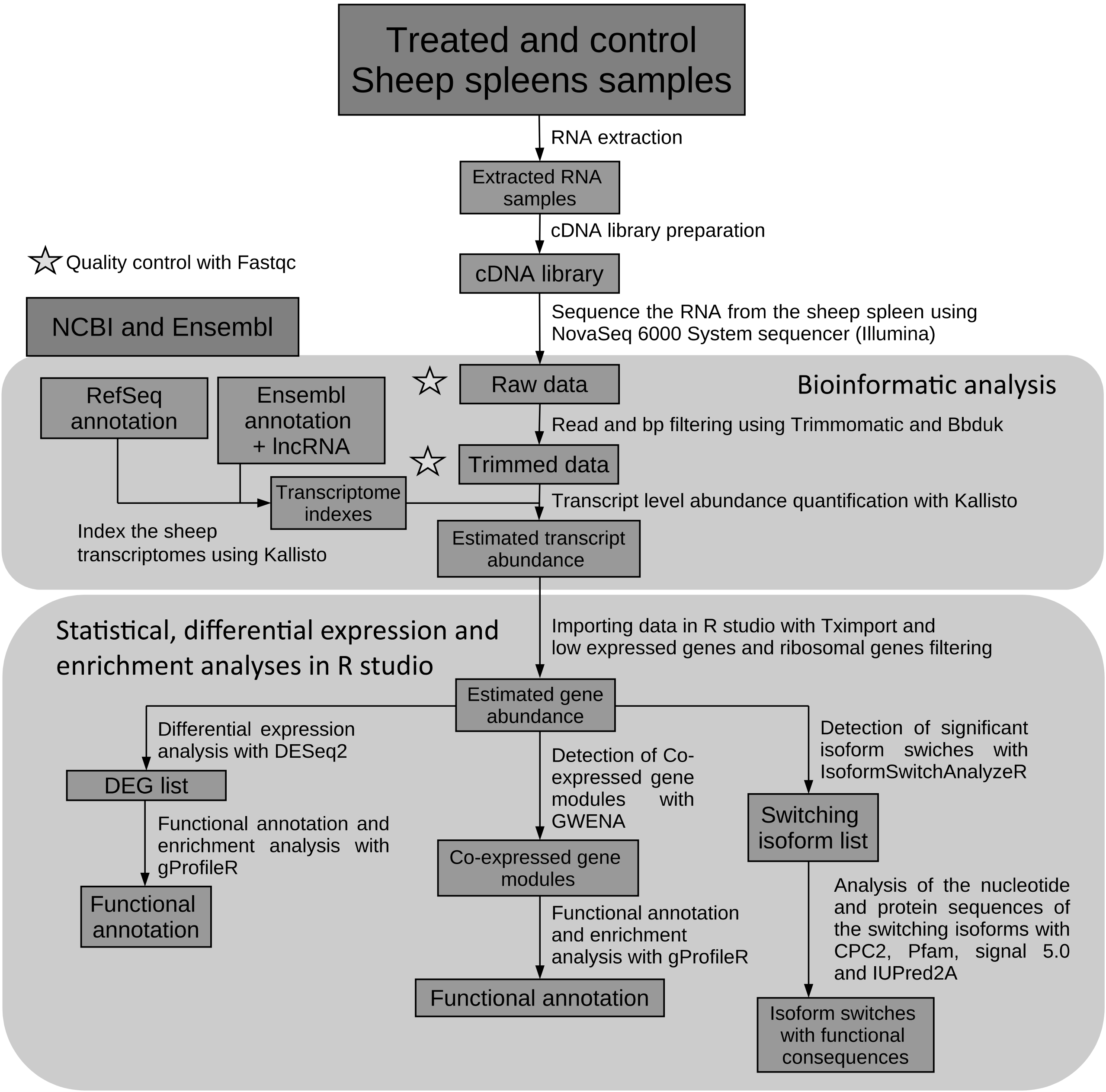

Supplementary figure 1. This pipeline shows the steps from tissue collection to RNA sequencing and the workflow followed by all samples for bioinformatic, statistical, differential expression and enrichment analyses in this work. The programs used in each step are also shown: Multiqc, Trimmomatic, Bbduk, Kallisto, STAR and R-studio (Tximport, DESeq2, Factoextra, Pheatmapg ProfileR and packages).

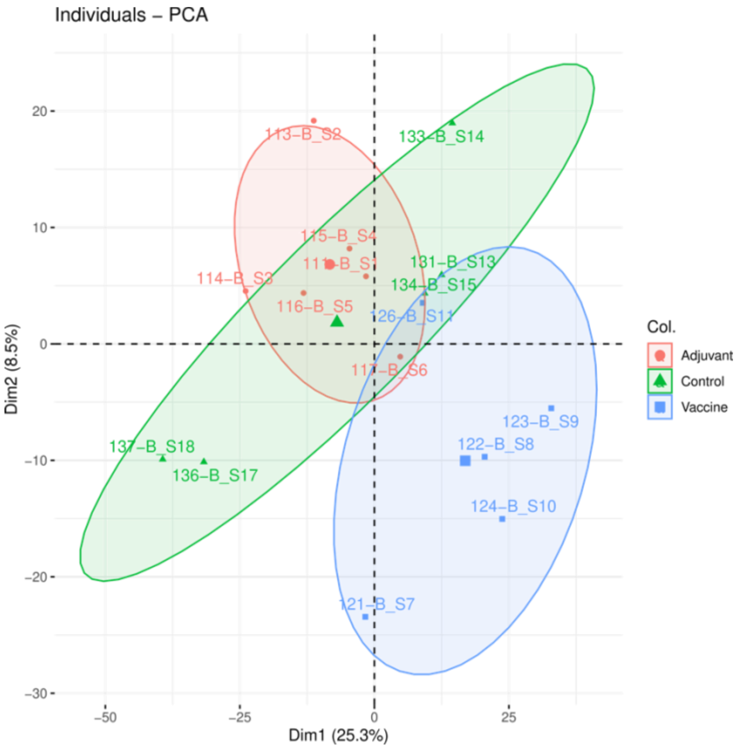

Supplementary figure 2: Principal component analysis (PCA) of the normalized (with DESeq2's median of ratios) and ln transformed expression in the RefSeq abundance set after outlier samples removal. The first and second principal components (ordered by variation proportion) are shown.

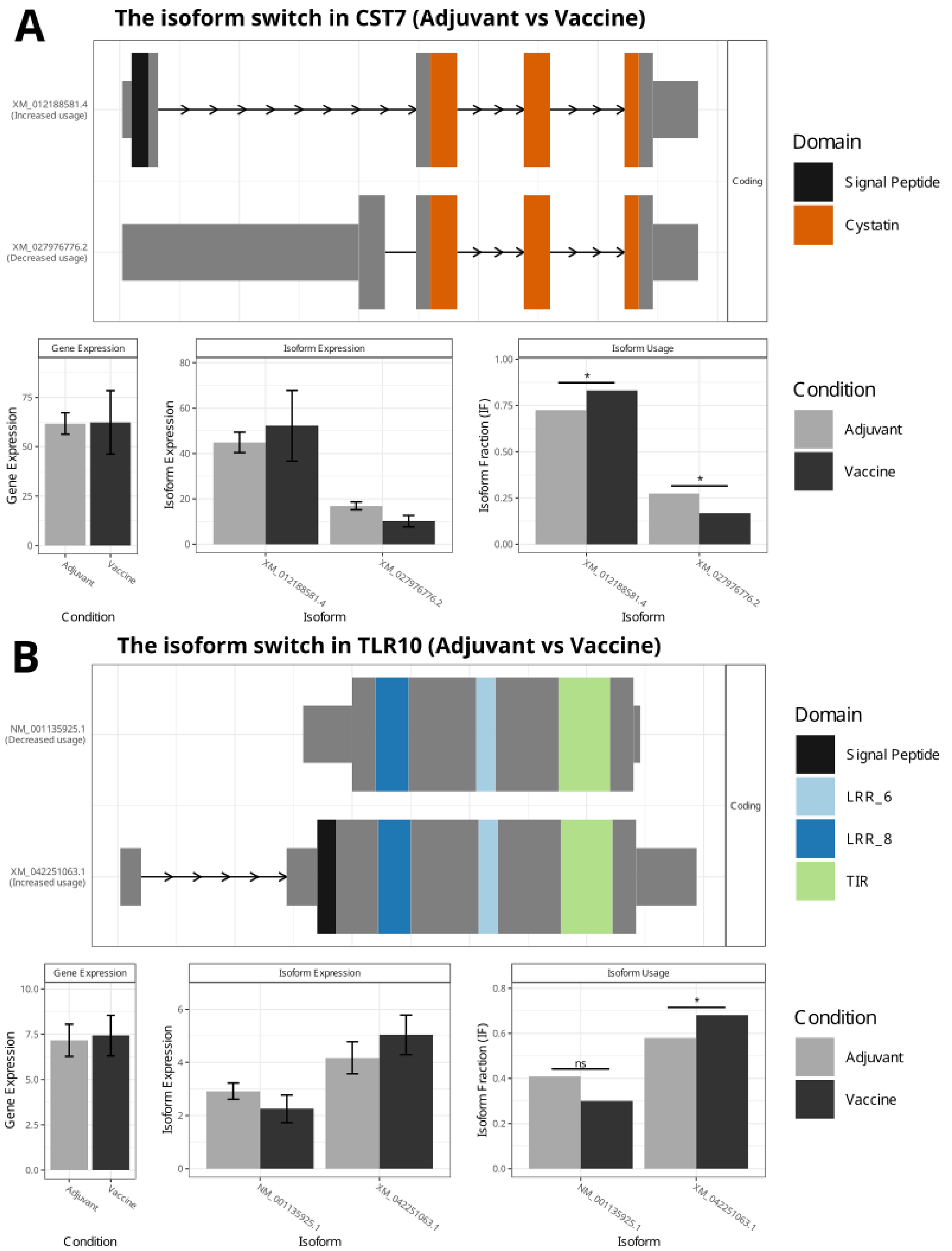

Supplementary figure 3: Structural analysis of all the differentially used isoforms for **A.** EPSTI1 and **B.** GON4L. The comparison of their gene expression, isoform expression and isoform fraction in Adjuvant and vaccine conditions is also showed for each gene.

1.2 Supplementary Table

Supplementary Table 1. The selected 18 samples for this study. The sample name and condition, the RNA integrity number (RIN) before the library preparation, the sequencing depth, the read counts and proportion after data preprocessing and the pseudoalignment rates obtained with Kallisto against the RefSeq transcriptome (Kallisto RefSeq) and Ensembl's extended transcriptome (Kallisto Ensembl).

Sample Name	Condition	RIN Quality Score	Raw reads	Filtered reads	Kallisto RefSeq	Kallisto Ensembl
111-B	Adjuvant	7.9	107846947	103311251 (95,79%)	43.84	33.87
113-B	Adjuvant	8.0	141336800	135169171 (95,64%)	40.59	31.52
114-B	Adjuvant	7.5	118298113	113070007 (95,58%)	36.42	28.04
115-B	Adjuvant	8.3	108244705	103243758 (95,38%)	42.23	32.84
116-B	Adjuvant	8.4	117900841	113181078 (96,00%)	39.57	29.92
117-B	Adjuvant	8.5	116794593	110759568 (94,83%)	45.03	34.82
121-B	Vaccine	8.6	107283962	101684980 (94,78%)	42.87	33.79
122-B	Vaccine	8.5	123631798	115821411 (93,68%)	50.55	39.63
123-B	Vaccine	8.6	76377675	70752357 (92,63%)	54.80	42.99
124-B	Vaccine	8.7	110854249	104081581 (93,89%)	51.98	41.14
126-B	Vaccine	8.1	105256030	99220057 (94,27%)	46.29	35.95
127-B	Vaccine	8.1	142268809	134846096 (94,78%)	40.59	32.12
131-B	Control	8.5	134754675	124994455 (92,76%)	48.23	37.37
133-B	Control	8.5	96693529	82975600 (85,81%)	49.13	39.05
134-B	Control	8.1	113795731	106467136 (93,56%)	46.46	36.30
135-B	Control	8.0	116693554	105574078 (90,47%)	33.97	28.74
136-B	Control	9.2	126239435	120634661 (95,56%)	35.28	27.07
137-B	Control	8.2	162015024	156535562 (96,62%)	33.60	25.12

Supplementary Tables captions

Supplementary Table 2. Results of the differential expression analysis of all the genes in A) Vaccine vs. Control comparison; B) Adjuvants vs. Control comparison; and C) Vaccine vs. Adjuvant comparison.

Supplementary Table 3. Correlation among diferentially expressed lncRNAs and the most proximal gene in a 100Kb window to search for cis-acting lncRNAs in A) Vaccine vs. Control; and B) Vaccine vs. Adjuvant.

Supplementary Table 4. Results from the enrichment analysis of the differentially expressed genes in A) Vaccine vs. Adjuvant comparison; and B) Vaccine vs. Control comparison.

Supplementary Table 5. A) List of all genes used for co-expression analysis and the module to which they belong; B) Enriched terms in all the genes of each detected module in the weighted co-expression network analysis; and C) List of hub genes in the two modules significantly correlated with the vaccine treatment (ME1 and ME3).

Supplementary Table 6. Results from the enrichment analysis of the hub genes from A) ME1 module; and B) ME3 module.
